# Patellar Tendon Properties and Unilateral Jump Performance in Junior Elite Volleyball Players With Patellar Tendinopathy

**DOI:** 10.1002/ejsc.70080

**Published:** 2025-11-08

**Authors:** Ra'ad M. Khair, Taija Finni, Timo Rantalainen, Mikko Häyrinen, Krista Tapaninaho, Miika Köykkä

**Affiliations:** ^1^ Faculty of Sport and Health Sciences University of Jyväskylä Jyväskylä Finland; ^2^ Finnish Institute of High‐Performance Sport KIHU Jyväskylä Finland

**Keywords:** countermovement jump, injury, jumper's knee, tendon properties, ultrasound

## Abstract

Athletes with patellar tendinopathy (PT) seem to have superior jumping performance compared to asymptomatic counterparts. However, previous studies have primarily assessed bilateral tasks which does not capture unilateral impairments. Hence, this study aimed to assess the patellar tendon properties and unilateral jump performance, and their associations in volleyball athletes with and without PT. Lower extremity injury assessment, patellar tendon properties using shear wave (SW) elastography and comprehensive jump performance analysis were performed. From 27 athletes (16 females) included in the study, 13 had unilateral PT. Three‐way ANOVA was used to evaluate the influence of PT. Differences between asymptomatic and symptomatic athletes were examined using simple contrasts. Moreover, we explored associations between patellar tendon properties and performance. Jump performance did not differ between or within asymptomatic and symptomatic athletes. In unilateral PT, the painful limb had lower SW velocity compared to the non‐painful limb with a mean difference of −1.7 m × s^−1^ (95% CI −3.32 to −0.013 m × s^−1^) and compared to asymptomatic athletes −1.4 m × s^−1^ (95% CI −2.59 to 0.20). Regardless of condition, patellar tendon cross sectional area (CSA) was negatively correlated with jump height in both sexes. In males, CSA correlated negatively with SW velocity (*r* = −0.55, *p* = 0.008), while in females, SW velocity was negatively correlated with countermovement jump unweighting duration (*r* = −0.45, *p* = 0.023) and peak braking phase power (*r* = −0.60, *p* = 0.001). Jumping performance was not different in volleyball athletes with unilateral PT from their asymptomatic counterparts. Athletes with inferior jumping performance had larger patellar tendon CSA while tendinopathic tendons had worse patellar tendon properties.

## Highlights

- A novel finding of this study was the correlation between SW velocity and duration of countermovement jump phases in females, suggesting that stiffer tendons allow shorter unweighting and longer propulsive phase.
- The data suggest that in tendinopathic tendons, CSA of the patellar tendon might be a good indicator of degenerative changes.
- For athletes in frequent jumping sports, monitoring of countermovement jump strategy could be complemented with duration of unweighting and propulsive phases since they may indicate better patellar tendon properties particularly in females. Future longitudinal studies are needed to further understand the progression of tendinopathy on patellar tendon structure and its effect on jumping performance.

## 1 Introduction

Patellar tendinopathy (PT) is one of the most prevalent conditions in high‐demand jumping sports (Ø. B. Lian et al. 2005). In elite volleyball players, the incidence of PT can reach up to 45% with men reporting twice the injury prevalence than women (Ø. B. Lian et al. 2005; Janssen et al. 2015; O. Lian et al. 1996). PT in junior volleyball athletes is associated with long‐term knee dysfunction, with approximately 20% retiring from competitive sport due to the condition (Visnes et al. 2024). Volleyball players might perform 250–300 jumps in a five‐set match, in addition to their weekly training where jumps are involved in most of the essential key skills (Hasegawa et al. 2008; Visnes et al. 2013). Such repeated stress on the patellar tendon, combined with insufficient recovery time, can accumulate damage and induce structural changes (Gisslèn et al. 2005; Gisslén and Alfredson 2005). Indeed, tendinopathy is associated with, for example, increased type III collagen content (Bank et al. 1999; Riley et al. 1994; Tom et al. 2009) and a larger cross‐sectional area (CSA) (Arya and Kulig 2010; Helland et al. 2013; Hjortshoej et al. 2024). The shift from collagen type I to type III may alter tendon mechanical properties, reducing stiffness and Young's modulus (Arya and Kulig 2010; Helland et al. 2013). In volleyball athletes with PT, lower mechanical stiffness and larger proximal CSA in the patellar tendon were observed in comparison to matched asymptomatic controls (Helland et al. 2013).

While some studies suggested that athletes with PT had superior countermovement jump (CMJ) performance compared to asymptomatic athletes (Ø. Lian et al. 1996, 2003), recent studies found no difference in CMJ and squat jump height between athletes with PT and matched controls (Helland et al. 2013; Ø. Lian et al. 2003). However, a greater difference between the two jumps was observed in the PT group (Helland et al. 2013), possibly indicating better use of the stretch‐shortening cycle (Bojsen‐Møller et al. 2005). There is also evidence that volleyball players with PT, show altered jump strategies in CMJ and drop jumps (DJ), compared to athletes without any PT history, indicated by greater work done (Ø. Lian et al. 2003), with no difference in jump height. While previous studies have focused on assessing bilateral jumping tasks in athletes with PT, unilateral impairments and altered jumping strategy could be expected in the injured limb.

Shear wave elastography (SWE) is a technique recently adapted that allow assessment of tendon mechanical properties (Bercoff et al. 2004; Haen et al. 2017; Khair et al. 2024; Sukanen et al. 2023). Tissue elasticity can be inferred from shear wave (SW) velocity generated by radiation pulses (Bernabei et al. 2020). While SWE has shown promising potential in diagnosing athletes with unilateral PT (Z. J. Zhang et al. 2014), studies have reported conflicting results. Some studies found higher SW elasticity in athletes with PT compared to asymptomatic athletes (Breda et al. 2020), or in patients with PT (Coombes et al. 2018). Conversely, (Dirrichs et al. 2016) reported lower SW elasticity in patients with PT. Thus, additional research can provide a better understanding of how PT alters patellar tendon properties, and their relationship with jump performance in athletes engaged in frequent jumping sports.

Therefore, the purpose of the study was to investigate patellar tendon properties and unilateral jump performance, and their relationships in volleyball athletes with and without unilateral PT. We hypothesized: (1) lower SW velocity and greater patellar tendon CSA in the painful limb compared to the non‐painful limb and to asymptomatic athletes; (2) no difference in jump height but altered jumping strategy in the painful limb compared to non‐painful limb and to asymptomatic athletes; and (3) SW velocity of the patellar tendon to be associated positively with the jump performance, whereas CSA and thickness to have a negative associations to both SW velocity and jump performance (Sprague et al. 2022).

## 2 Materials and Methods

A convenience sample of 27 volleyball athletes (16 females, 11 males; mean ± SD age: 16.9 ± 0.8 years, height: 184.1 ± 10.1 cm, body mass: 79.1 ± 11.3 kg) was recruited from the National Volleyball Training Centre based in Kuortane Sport Institute during a yearly medical check in 2024. Inclusion criterion was active participation in competitive sport at a national level Tier 3 or higher (McKay et al. 2021). Ethical approval was obtained from the Human Sciences Ethics Committee of the University of Jyväskylä (Diary No. 504/13.00.04.00/2024) and all study procedures were conducted according to the Declaration of Helsinki. Participants received consent forms, and detailed information about the measurement protocol and were instructed to avoid vigorous exercises 24 h prior to data collection. All athletes reported their right arm as the preferred striking arm.

### 2.1 Clinical Examination

Participants went through a standardised check‐up for lower extremity injuries. Sports medicine physician conducted knee examination, following the diagnostic criteria for jumper's knee: history of pain localized to the lower patellar pole in connection with athletic activity, and distinct palpation tenderness corresponding to the painful area (Cook et al. 2000). Previous jumper's knee was diagnosed based on history alone. To assess the severity of the condition, athletes evaluated patellar tendon during the previous week with Visual Analogue Scale 0–10. All diagnosed athletes reported knee pain within the month preceding clinical assessment, with four athletes experiencing episodic symptoms for over 4 years. Among asymptomatic athletes at the time of examination, only one reported having experienced knee pain during the previous year. Detailed information for both symptomatic and asymptomatic athletes is provided in Table 1. It should be noted that athletes with acceptable pain levels (≤ 4 out of 10) were able to continue their training activities.

**TABLE 1:** Descriptives, duration of symptoms, and average years of play of symptomatic and asymptomatic athletes.

	Age	Body mass index	Symptoms duration	Average years of play
Symptomatic athletes	16.8 (0.5)	22.2 (2.9)	3.2 (2.7) years	5.4 (2.1)
Asymptomatic athletes	16.9 (0.9)	26.1 (5.5)	—	4.7 (2.0)

### 2.2 Testing Protocol

Upon arriving at the testing facility, participants were positioned supine with knees flexed at 30°, supported by a bolster. The patellar inferior pole was identified using a 38 mm ultrasound probe (Aixplorer Supersonic Imagine, *v*. 12.3.1 Aix‐en‐Provence, France, 38 mm, SL10‐2) and marked on the skin. Two extended field of view images were taken from the midline of the tendon starting from the tibial tuberosity to the inferior pole of the patella, and two still B‐mode images on the axial plane 1 cm distal to the inferior pole of the patella to estimate the tendon CSA (Sprague et al. 2022). Then, SWE videos were taken with the proximal edge of the probe placed 1 cm distal to inferior pole of the patella. When a clear image of the patellar tendon was captured, two 5‐s videos were recorded and saved for offline analysis (Bercoff et al. 2004). After ultrasound measurements, the bolster was removed, and the shank length was measured from the midline of the knee joint to the distal edge of the lateral malleolus with participant lying in supine position.

**FIGURE 1:**
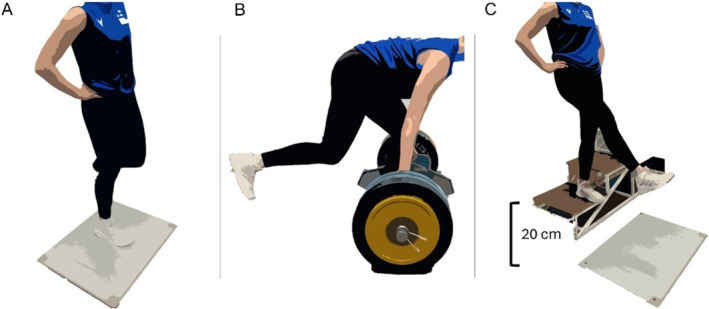
Illustrates the jumping and the unilateral hex bar pull (UIHBP) tests: (A) unilateral countermovement jump starting position with hands placed on waist, (B) UIHBP at a knee angle of 115° flexion (C) unilateral drop jump from a 20‐cm box.

Subsequently, participants performed a 5‐min aerobic warm‐up, followed by submaximal DJs and CMJs. Then, three maximal unilateral CMJs and DJs (off a 20‐cm box), respectively, were performed with hands placed on waist. The rest intervals were 1‐min. In CMJs, participants stood still for the initial 3 seconds of the data collection, then rapidly squatted to their preferred depth and immediately jumped as high as possible (Figure 1). In DJs, participants stepped off a box, landed with one leg with knees and hips as straight as possible, and then jumped as fast and high as possible. After landing, they stabilised in an upright posture for approximately 3 seconds.

After 2 min of rest, participants were visually introduced to the unilateral hex bar pull test (UIHBP). This test was performed following McPhail et al. (2021) protocol using a 160 kg loaded hex bar with the handle positioned 33.5 cm from the floor and the knee angle at 115° flexion (McPhail et al. 2021). For taller individuals, blocks were placed under the weights to raise the bar to ensure correct knee angle. Participants first performed bilateral hex bar deadlift from the ground. Once familiar with the test, the uninvolved limb was raised backward, and the participant completed a series of warm up trials from 70% to 90% of the self‐estimated maximal effort. After a 3‐min rest, the participants were instructed to ‘pull against the bar and push into the ground as hard as possible’, exerting maximal force for 4 s. Each limb was tested 3 times with 60‐s rest between trials.

### 2.3 Ultrasound Data Analysis

Thickness and CSA of the patellar tendon were analysed from B‐mode images using ImageJ (1.44b, National Institutes of Health). Thickness was measured at the thickest point of the extended field of view image as in Sprague et al. (2022).

Patellar tendon SWE was assessed using the same 38 mm (SL10‐2) probe, using a custom preset (penetration mode, smoothing level 5, persistence off, opacity 100%, SWE elasticity range 0–16.3 m × s^−1^). SWE videos were analysed using semi‐automated processing software (ElastoGUI, University of Nantes, France) in MATLAB by a trained research intern who was blinded to the existence of PT symptoms. The analysed region was selected to cover the largest area possible, and limits for saturation and void were set at < 3% and < 0.1%, respectively. The average (SD) areas analysed with SW were 0.64 cm^2^ (0.15) and 0.62 cm^2^ (0.16) for the left and right limbs, respectively. Saturation on average was 0.13% and 0.10% in the left and limbs, respectively. Shear wave (SW) measurements are reported as SW velocity (m × s^−1^). Reliability for the single assessor was tested with healthy population (*n* = 6), carried out during one laboratory visit with approximately 15 min between two trials. Intra‐rater reliability of SWE imaging resulted with intra‐class correlation coefficient (ICC_3_ˌ_1_) 0.94 (95% CI 0.59–0.99) and coefficient of variation (CV) of 9.5%. The standard error of measurement (SEM), and minimum detectable change (MDC) of the measurements were 0.63 m × s^−1^ and 1.75 m × s^−1^, respectively.

### 2.4 Jump and UIHBP Analysis

CMJs, DJs, and UIHBPs were performed on a force plate (1000 Hz, HUR labs, Finland) and data collected via the Coachtech online system (University of Jyväskylä, Finland) (Ohtonen et al. 2015). CMJ phases—unweighting, braking, and propulsion—were analysed as per (J. J. McMahon et al. 2018), with calculations for phase durations, time to take‐off, jump height, reactive strength index (RSI_mod_) (jump height/time to take‐off), countermovement depth (height‐normalised), and peak power during braking and propulsion phases (J. J. McMahon et al. 2017). DJ phases were divided into braking and propulsion, with calculations for phase durations, contact time, jump height, RSI (jump height/contact time), peak landing force, and peak power during braking and propulsion phases following (J. J. McMahon et al. 2021). From UIHBP, absolute peak force and body weight‐normalised relative peak force were extracted. Previous studies have reported good to excellent test‐retest reliability for both the UIHBP test and CMJ assessments (McPhail et al. 2021; Fahey et al. 2024; Bishop et al. 2021). The test‐retest reliability of the DJ test was evaluated with nine recreational and national level athletes (*n* = 9), over two separate visits 1 week apart, yielding an average ICC_2,1_ of 0.81 and CV of 6.1%, with ICC_2,1_ values ranging from 0.70 to 0.93.

### 2.5 Statistical Analysis

The level of significance was set at *p* < 0.05, and descriptive data are reported as mean (SD). Skewness and kurtosis of the data were checked to determine data normality. A three‐way repeated measures ANOVA was performed to examine differences between limbs (painful from symptomatic athletes and non‐painful for asymptomatic athletes) versus (non‐painful from symptomatic athletes and non‐painful for asymptomatic athletes) as within‐subject, with unilateral pain (yes, no) and sex (M, F) as between‐subject factors. The primary interest of the analysis was in the interaction effects of unilateral pain and unilateral pain*sex on the measured variables. Asymptomatic athletes with bilaterally pain‐free limbs were randomly assigned to ensure equivalent proportion of right/left limbs between athletes with unilateral pain and those without pain. Significant three‐way interactions were followed by two‐way analysis and Tukey's post‐hoc. Due to the complexity of the three‐way repeated measures model, the statistical power may have been reduced. Hence, additional simple contrasts averaged over the levels of sex were performed to compare between asymptomatic and symptomatic athletes. Relationships between properties of the patellar tendon and jump performance were explored using Pearson's correlation coefficient. If bivariate normality was not met, Spearman's rho was used. Data from both limbs were combined for the analysis. Given the differences between males and females in tendon properties and jump performance, the correlations were computed separately between sexes (J. J. McMahon et al. 2017; G. McMahon and Cook 2024). All statistical tests were performed using JASP (JASP 0.18, Netherlands). Intra rater reliability of SWE measurements was calculated using ICC_3,1_, CV, SEM, and MDC (Hopkins 2000), while DJ test‐retest reliability was evaluated using ICC_2,1_ and CV.

## 3 Results

Out of the 27 athletes, 14 (52%) were diagnosed with patellar tendinopathy, and one athlete with bilateral PT was excluded from the analysis. Among the 11 male and 15 female athletes, 8 (72%) and 5 (33%), respectively, experienced unilateral patellar tendon pain. The median (IQR) Visual Analogue Scale score of the unilateral pain group was 5.0 (2.5) points. Four athletes failed to attend the jump assessment, and two athletes could not perform the UIHBP due to pain. Table 2 shows the descriptive data of the results.

**TABLE 2:** Descriptive data of the measured variables of symptomatic and asymptomatic athletes categorized by sex. In asymptomatic athletes, the legs were randomly assigned to match the right and left legs corresponding to the proportions in symptomatic athletes.

	Asymptomatic athletes (*n* = 13)				Symptomatic athletes (*n* = 13)			
	Male (*n* = 3)		Female (*n* = 10)		Male (*n* = 8)		Female (*n* = 5)	
	Non‐painful	Non‐painful	Non‐painful	Non‐painful	Painful	Non‐painful	Painful	Non‐painful
SW velocity (m × s^−1^)	11.4 (1.8)^a^	10.7 (2.1)	10.4 (1.8)^a^	10.1 (1.8)	8.7 (1.2)^b^	11.2 (1.2)	9.9 (1.6)^b^	10.2 (2.0)
Patellar tendon CSA (mm^2^)	68.4 (1.8)	74.7 (16.6)	67.0 (22.3)	63.1 (16.1)	100.2 (26.4)	91.9 (21.8)	65.5 (11.8)	63.5 (21.8)
Thickness (mm)	4.6 (0.4)	4.8 (0.3)	4.3 (0.8)	4.0 (0.7)	4.9 (0.4)	5.1 (0.9)	4.6 (0.6)	4.2 (0.8)
UIHBP (*N*/kg)	2.1 (0.4)	2.1 (0.2)	1.9 (0.3)	2.0 (0.3)	2.2 (0.4)	2.1 (0.3)	2.0 (0.2)	2.1 (0.1)
Countermovement jump								
Height (cm)	23.8 (3.2)	22.5 (4.3)	11.7 (2.6)	12.2 (3.9)	21.4 (5.7)	20.5 (6.5)	16.9 (1.9)	16.9 (1.4)
Depth (cm)	−28.2 (7.5)	−22.1 (2.8)	−17.3 (4.2)	−17.7 (2.0)	−21.7 (5.6)	−23.7 (6.8)	−22.6 (2.0)	−22.7 (6.7)
Depth (%height)	−0.16 (0.05)	−0.12 (0.01)	−0.09 (0.02)	−0.10 (0.03)	−0.12 (0.03)	−0.13 (0.03)	−0.12 (0.02)	−0.12 (0.05)
Propulsive duration (ms [relative])	339.1 (40.6) [39.8 (3.1)]	320.0 (24.6) [40.6 (2.7)]	303.3 (67.0) [40.4 (6.1)]	304.4 (69.4) [39.7 (7.1)]	303.1 (31.4) [37.2 (3.3)]	297.0 (33.3) [37.9 (3.4)]	297.0 (52.5) [38.1 (0.7)]	299.8 (73.7) [38.2 (2.5)]
Unweighting duration (ms [relative])	314.6 (50.5) [37.0 (6.2)]	284.4 (27.5) [36.1 (3.6)]	294.3 (107.3) [37.7 (9.1)]	316.3 (130.2) [390 (10.3)]	333.1 (93.5) [39.9 (1.7)]	310.1 (64.6) [38.6 (4.4)]	315.2 (71.5) [39.9 (1.7)]	292.1 (42.7) [37.4 (2.7)]
Braking duration (ms[relative])	197.0 (31.5) [23.2 (3.1)]	184.0 (10.1) [23.3 (1.0)]	164.7 (32.2) [21.9 (3.4)]	163.4 (40.9) [21.2 (3.6)]	188.9 (26.5) [23.1 (1.7)]	185.3 (21.9) [23.6 (1.0)]	171.9 (33.1) [21.9 (1.2)]	190.9 (38.1) [24.5 (1.0)]
Peak power braking (*N*/kg)	−12.2 (3.1)	−11.4 (2.2)	−8.8 (3.4)	−8.3 (2.2)	−10.5 (1.9)	−10.8 (2.4)	−9.5 (2.9)	−8.7 (2.1)
Peak power propulsive (*N*/kg)	36.9 (2.6)	35.6 (5.3)	24.9 (3.9)	25.7 (4.5)	34.9 (5.9)	34.8 (2.2)	30.5 (2.5)	30.8 (2.2)
RSI_mod_	0.3 (0.04)	0.3 (0.05)	0.16 (0.04)	0.16 (0.05)	0.3 (0.1)	0.3 (0.1)	0.2 (0.1)	0.2 (0.1)
Drop jump								
Height (cm)	20.1 (1.2)	17.8 (4.6)	10.6 (3.2)	10.3 (3.4)	16.2 (5.2)	16.1 (5.4)	13.8 (1.8)	15.2 (2.0)
Propulsive duration (ms [relative])	177.8 (6.3) [55.1 (2.8)]	173.0 (13.7) [54.4 (0.24)]	174.8 (31.9) [55.6 (2.5)]	173.5 (27.8) [55.6 (2.3)]	163.0 (25.2) [53.9 (1.7)]	168.9 (35.1) [53.6 (1.1)]	170.4 (31.3) [53.8 (2.5)]	165.5 (20.4) [53.3 (1.8)]
Braking duration (ms[relative])	145.9 (21.1) [44.9 (2.8)]	144.9 (10.6) [45.6 (0.2)]	139.3 (22.6) [44.4 (2.5)]	138.8 (25.2) [44.4 (2.3)]	139.9 (24.3) [46.1 (1.8)]	146.6 (30.3) [46.4 (1.2)]	145.5 (16.7) [46.2 (2.5)]	144.7 (7.5) [46.7 (1.8)]
Peak power braking (*N*/kg)	−50.2 (12.5)	−45.1 (13.6)	−46.8 (10.5)	−47.5 (12.9)	−48.5 (9.7)	−45.3 (1.0)	−40.9 (6.2)	−48.6 (1.0)
Peak power propulsive (N/kg)	40.5 (0.5)	37.0 (6.4)	28.1 (4.7)	27.6 (5.2)	37.2 (7.4)	37.5 (8.1)	33.4 (2.4)	35.1 (3.6)
Peak landing force (%body weight)	367.9 (55.0)	342.2 (55.5)	366.0 (61.1)	375.1 (74.2)	375.3 (30.2)	354.9 (45.3)	333.4 (19.0)	351.5 (14.2)
RSI	0.62 (0.02)	0.56 (0.2)	0.35 (0.1)	0.34 (0.1)	0.55 (0.19)	0.5 (0.1)	0.44 (0.1)	0.5 (0.1)

^a^ denotes statistical differences from simple contrasts analysis between symptomatic and asymptomatic athletes randomly matched limbs. It should be noted that statistical analyses were conducted using data from both sexes in the ANOVA models.

^b^ denotes statistical differences within symptomatic athletes between painful and non‐painful limbs.

### 3.1 Tendon Properties, UIHBP and Jump Performance Interactions With PT

**FIGURE 2:**
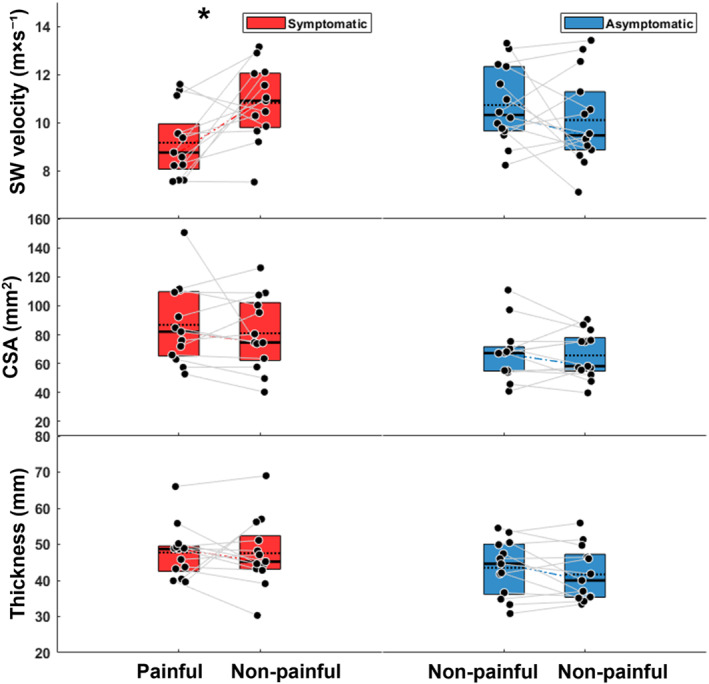
Box plots showing means (dotted line), and median (solid line) ± SD of patellar tendon SW velocities (top), CSA (middle) and thickness (bottom) between symptomatic athletes with unilateral pain (red) and asymptomatic (blue). In asymptomatic athletes, the legs were randomly assigned to match the right and left legs corresponding to the proportions in symptomatic athletes. *Post‐hoc difference between limbs (*p* < 0.05).

Three‐way repeated measures ANOVA was performed to evaluate the influence of unilateral pain and sex on the measured variables. There was an interaction of unilateral pain on SW velocity (*F* (1–23) = 4.7, *p* = 0.041), confirmed by the two‐way analysis (*p* = 0.018). Post‐hoc analysis showed differences between the painful and non‐painful limbs in the PT group with a mean difference −1.7 m × s^−1^ (95% −3.32 to −0.013 m × s^−1^, *p* = 0.041) (Figure 2). No interaction effects or main effects of unilateral pain were observed on the patellar tendon thickness, CSA, UIHBP, nor any of the jump test metrics either between or within athletes. Main effects of sex on the measured variables can be found in Supporting Information S1.

### 3.2 Simple Contrast Between Symptomatic and Asymptomatic Athletes

Symptomatic athletes had lower SW velocity in the injured limb compared to randomly matched limbs of asymptomatic athletes with mean difference of −1.4 m × s^−1^ (95% CI −2.59 to 0.20, *p* = 0.023). There were no differences between symptomatic and asymptomatic athletes in any of the performance metrics or patellar tendon properties.

### 3.3 Relationship Between Patellar Tendon Properties and Performance Characteristics

**FIGURE 3:**
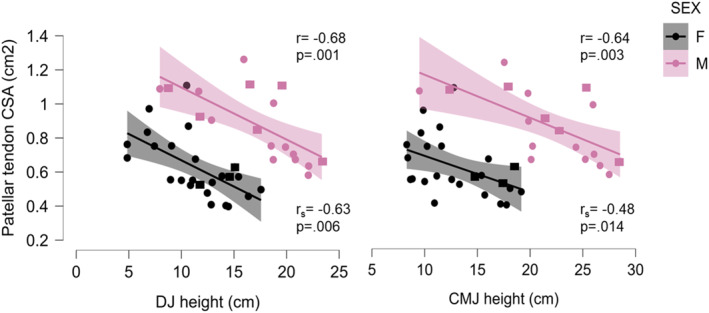
Scatterplots and regression lines of the correlations between cross sectional area of the patellar tendon and drop jump heigh (left) and countermovement jump height (right) for males and females. Asymptomatic athletes are represented by circles, and symptomatic athletes by squares. It should be noted that sex had a main effect of countermovement jump and drop jump height *p* < 0.005.

In both sexes CMJ and DJ height correlated negatively with CSA of the patellar tendon (Figure 3). In males, jump metrics correlated predominantly with tendon CSA. In females, jump metrics correlated with tendon CSA, thickness and SW velocity, and UIHBP strength correlated with tendon CSA. All correlations between patellar tendon properties and jump performance variables are presented in Table 3. For clarity, only variables with significant correlations are mentioned.

**TABLE 3:** Correlations between tendon properties and performance characteristics, number of data points between patellar tendon properties and unilateral hex bar pull, countermovement jump, and drop jump tests were *n* = 43, 43, 43, and 44 when pooled together, *n* = 21, 23, 25, 26 for females, and *n* = 22, 20, 18, 18 for males, respectively.

	Patellar tendon CSA (cm^2^)		Thickness (cm)		SW velocity (m × s^−1^)	
	Males	Females	Males	Females	Males	Females
Patellar tendon CSA	—	—	*r* = _−_0.25, *p* = 0.28	***r* =** _**−**_ **0.47, *p* = 0.012**	***r* = −0.55, *p* = 0.008**	*r* = 0.10, *p* = 0.57
UIHBP	*r* = −0.40, *p* = 0.06	***r***_***s***_ **= −0.75, *p* = 0.001**	*r*_*s*_ = 0.22, *p* = 0.38	*r*_*s*_ = −0.43, *p* = 0.051	*r* = 0.04, *p* = 0.84	*r*_*s*_ = −0.19, *p* = 0.40
Countermovement jump (CMJ)						
Height	***r* = −0.64, *p* = 0.003**	***r***_***s***_ **= −0.48, *p* = 0.014**	*r*_*s*_ = −0.08, *p* = 0.76	***r***_***s***_ **= −0.42, *p* = 0.038**	*r* = 0.21, *p* = 0.39	*r*_*s*_ = 0.02, *p* = 0.88
Depth	***r* = 0.53, *p* = 0.02**	*r*_*s*_ = 0.36, *p* = 0.08	*r*_*s*_ = −0.30, *p* = 0.22	***r***_ **=** _ **0.40, *p* = 0.046**	*r* = −0.37, *p* = 0.13	*r* = −0.10, *p* = 0.61
Relative propulsive duration	*r* = 0.02, *p* = 0.93	*r*_*s*_ = 0.31, *p* = 0.14	*r*_*s*_ = −0.15, *p* = 0.54	*r* = −0.22, *p* = 0.28	*r* = −0.01, *p* = 0.98	***r***_ **=**_ **0.60,** ***p* = 0.001**
Relative unweighting duration	*r* = −0.02, *p* = 0.87	*r*_*s*_ = −0.26, *p* = 0.21	*r*_*s*_ = −0.24, *p* = 0.34	*r*_*p*_ = −0.23, *p* = 0.26	*r*_*p*_ = 0.04, *p* = 0.84	***r* = −0.45, *p* = 0.023**
Relative braking duration	*r* = 0.09, *p* = 0.71	*r* = 0.31, *p* = 0.13	***r***_***s***_ **= 0.51, *p* = 0.03**	*r*_*s*_ = 0.40, *p* = 0.09	*r* = −0.13, *p* = 0.61	*r* = 0.34, *p* = 0.009
Peak power braking	***r* = 0.51, *p* = 0.03**	*r*_*s*_ = 0.09, *p* = 0.66	*r*_*s*_ = 0.02, *p* = 0.95	*r* = −0.12, *p* = 0.57	*r* = −0.28, *p* = 0.26	***r* = −0.60, *p* = 0.001**
Peak power propulsive	***r* = −0.62, *p* = 0.006**	*r*_*s*_ = −0.33, *p* = 0.11	*r*_*s*_ = −0.03, *p* = 0.90	*r* = −0.28, *p* = 0.17	*r* = −0.22, *p* = 0.38	*r* = 0.19, *p* = 0.34
RSI_mod_	***r* = −0.62, *p* = 0.006**	*r*_*s*_ = −0.18, *p* = 0.38	*r*_*s*_ = 0.02, *p* = 0.55	***r* = −0.50,** ***p* = 0.011**	*r* = −0.23, *p* = 0.36	*r* = 0.12, *p* = 0.56
Drop jump (DJ)						
Height	***r* = −0.68, *p* = 0.001**	*r*_*s*_ = **−0.63, *p* = 0.005**	*r*_*s*_ = 0.13, *p* = 0.61	***r* = −0.50,** ***p* = 0.009**	*r*_*p*_ = 0.35, *p* = 0.14	*r* = 0.11, *p* = 0.58
Peak landing force	*r*_*s*_ = −0.24, *p* = 0.34	*r*_*s*_ = −0.37, *p* = 0.06	*r*_*s*_ = −0.38, *p* = 0.12	***r***_***s***_ **= 0.45, *p* = 0.021**	*r*_*s*_ = −0.24, *p* = 0.34	*r*_*s*_ = −0.12, *p* = 0.55
Peak power braking	*r* = 0.26, *p* = 0.28	***r***_***s***_ **= 0.40, *p* = 0.041**	*r*_*s*_ = 0.42, *p* = 0.08	***r* = 0.48,** ***p* = 0.014**	*r* = −0.16, *p* = 0.52	*r* = −0.16, *p* = 0.42
Peak power propulsive	***r***_***s***_ **= −0.54, *p* = 0.02**	***r***_***s***_ **= −0.61, *p* = 0.001**	*r*_*s*_ = −0.02, *p* = 0.93	***r* = −0.45,** ***p* = 0.021**	*r*_*s*_ = 0.45, *p* = 0.05	*r* = 0.05, *p* = 0.81
RSI	***r***_***s***_ **= −0.53, *p* = 0.02**	***r***_***s***_ **= −0.67, *p* = 0.002**	*r*_*s*_ = −0.17, *p* = 0.49	***r* = −0.52,** ***p* = 0.006**	*r*_*s*_ = 0.29, *p* = 0.23	*r* = 0.03, *p* = 0.86

*Note:* Pearson's correlation coefficient is denoted as (*r*), while Spearman's rho as (*r*_*s*_). Bolded values indicate significant correlations *p* < 0.05.

## 4 Discussion

This study assessed properties of the patellar tendon and unilateral jump performance in volleyball athletes with and without unilateral PT. As hypothesized, SW velocity was lower in painful limbs of symptomatic athletes compared to their non‐painful limbs and the limbs of asymptomatic athletes. Partly confirming the hypothesis, there were no differences in jump metrics between asymptomatic and symptomatic athletes or between limbs in athletes with unilateral PT. As hypothesised, patellar tendon structural measures were associated with various lower limb performance metrics across the sample. This suggests that jumps and isometric strength may reflect structural differences in patellar tendons. A novel finding of this study was the correlation between SW velocity and CMJ phase durations and braking power in females, suggesting that stiffer tendons are linked to more powerful braking.

### 4.1 Patellar Tendon Properties and Performance Characteristics

After accounting for sex, we found no differences in jump metrics or isometric strength between asymptomatic and symptomatic athletes, nor between symptomatic and asymptomatic limbs in athletes with PT. These findings partly align with previous studies (Helland et al. 2013; Ø. Lian et al. 2003) that reported no differences in jump heights between players with PT and those without PT history. However, Lian et al. (2003) found that players with PT performed more work in vertical jumps, suggesting a different jump strategy (Ø. Lian et al. 2003). It is important to note that these results are not entirely comparable since in Lian et al. (2003) utilised bilateral jumps, while this study focused on unilateral tests. Despite PT symptoms, athletes appear to be able to perform individual jumping tasks unilaterally without significant changes in performance.

SW velocity was lower in the patellar tendon of the painful limb compared to the non‐painful limb in symptomatic athletes and asymptomatic athletes. Our findings align with Dirrichs et al. (2016) who reported lower patellar tendon elasticity in injured limbs. In contrast, previous studies found a greater elastic modulus in patients with unilateral PT compared to the non‐painful limb (Z. J. Zhang et al. 2014) and asymptomatic control group (Z. J. Zhang et al. 2014; Coombes et al. 2018). However, different data analysis methods were used; our study covered an average of 63 mm^2^ of tendon area 1 cm below the patella's inferior pole, whereas Dirrichs et al. (2016) focused on the most rigid area with a 1 mm diameter, and Z. J. Zhang et al. (2014) assessed a small region with hypoechoic degenerative changes (Z. J. Zhang et al. 2014). Therefore, methodological considerations are advised when comparing SWE results between studies.

The thickness and CSA of the patellar tendon in the painful limb did not differ from that in the non‐painful limb or the asymptomatic group. This contrasts with previous studies reporting larger CSA in athletes with PT compared to asymptomatic athletes (O. Lian et al. 1996; Helland et al. 2013). Theoretically, a larger CSA could ameliorate degraded material quality in pathological tendons, although structural alterations may not correspond to symptoms (e.g., Sprague et al. 2022). Tendinopathic tendons typically show substantial collagen disarray and hypoechoic areas (O. Lian et al. 1996), accompanied by increased proteoglycans and fluid content (Magnusson and Kjaer 2019), which might lead to a greater CSA. We observed that in males, a larger CSA was associated with lower SW velocity, but not with thickness. A larger CSA may be a favourable adaptation in conjunction with muscle adaptation to loading in healthy individuals, while in tendinopathic tendons, a larger CSA might serve as a compensatory mechanism for reduced intrinsic material properties (Couppé et al. 2021).

### 4.2 Relationship Between Patellar Tendon Properties and Performance Characteristics

SW velocity in females was associated with CMJ unweighting and propulsion relative durations and peak braking phase power, indicating that patellar tendon elasticity may affect females' jumping strategy. The results indicate that females with higher patellar tendon stiffness—assessed with SWE—spend proportionally less time during the unweighting phase, achieve lower (more negative) braking power, and spend proportionally more time in the propulsion phase. However, no associations were observed in males, which could be partially explained by the sex differences during jumping, where males utilize less recoil energy from the stretching phase compared to females (Komi and Bosco 1978).

We found that larger tendons associated with lower jump height in DJ and CMJ. This is in concordance with Sprague et al. (2022), who reported that larger CSA of the patellar tendon was associated with lower jump height in individuals with PT. However, such a relationship was not observed in healthy football players (Murtagh et al. 2018). In our study, larger CSA was linked to lower peak power and a lower RSI during DJ in both sexes. This suggests impaired neuromuscular function, specifically a decrease in the efficiency of the stretch‐shortening cycle during DJ. Similar observations have been previously made between DJ RSI and CSA in healthy recreational male endurance runners, but not in females (Rubio‐Peirotén et al. 2021). The findings of this study should be interpreted with caution, as the participants are in the maturation phase and may be more susceptible to developing tendinopathy. This increased susceptibility is likely due to non‐uniform adaptation of muscle and tendon at this age, along with low collagen formation in the patellar tendon, as tendon turnover primarily occurs within the first 15 years of life (Mersmann et al. 2017; C. Zhang et al. 2020).

Larger CSA in males was associated with higher (less negative) braking phase power and lower (less positive) propulsion phase power. Thus, athletes with larger CSA seem to have lower eccentric and concentric power capabilities, potentially explaining the negative association with jump height. Additionally, lower RSI_mod_ was associated with larger CSA in males, but since no correlations with phase durations were found, it is likely due to lower jump height. In females, thicker patellar tendons were associated with lower performance in CMJ, DJ and UIHBP. In males, thicker tendons were associated only with CMJ relative braking duration, indicative of a slightly different jump strategy. Variability between sexes in the associations of tendon properties and jumping performance is not surprising, given the documented differences in adaptability, morphological and physiological response to tendon loading (G. McMahon and Cook 2024; Magnusson et al. 2007; Sullivan et al. 2009) and jumping performance between sexes (J. J. McMahon et al. 2017).

### 4.3 Limitations

This study has some limitations. Firstly, this study used a convenience sample during annual medical screenings which resulted in unequal representation of sexes in the unilateral pain group (Janssen et al. 2015). This imbalance may have limited the statistical power of the analyses and generalizability of the findings. Secondly, while SWE seems to be a reliable tool for assessing tendon properties, it operate under the assumption that the measured tissues are linearly elastic, homogenous and isotropic, which are not fully met (Bercoff et al. 2004). Lastly, echo intensity was not included in the assessment of patellar tendon properties. Incorporating echo intensity might have provided additional insights into tendon tissue quality, particularly in the evaluation of hypoechogenic tendons, which are commonly observed in cases of PT (Hjortshoej et al. 2024).

## 5 Conclusion

Junior elite volleyball athletes with unilateral PT appear to be capable of performing unilateral jumping tasks without significant alterations in performance. Additionally, jumping strategy did not differ from that of healthy counterparts. In symptomatic athletes, the SW velocity of the painful tendon—a surrogate measure of tendon stiffness—was lower compared to the non‐painful tendon and asymptomatic athletes. While tendon CSA correlates positively with muscle strength across different healthy athletes (Arampatzis et al. 2013), in athletes with high jumping demand the tendon CSA was negatively associated with jump performance.

## Funding

This work was supported by Academy of Finland (ACHILLES, grant #355678/Taija Finni). Miika Köykkä’s work was partially supported by the Kuortane Olympic Training Centre. The funding organizations had no role in collection, analysis and interpretation of the data, or publication.

## Ethics Statement

Ethical approval was obtained from the Human Sciences Ethics Committee of the University of Jyväskylä (Diary No. 504/13.00.04.00/2024) and all study procedures were conducted according to the Declaration of Helsinki. All participants signed informed consent prior to participation. Athletes or members of public were not involved in the design or interpretation of this research.

## Conflicts of Interest

The authors declare no conflicts of interest.

## Supporting information


Supporting Information S1

## Data Availability

The data that support the findings of this study are available upon reasonable request from the authors.
