# Supplementary material for: Patellar Tendon Properties and Unilateral Jump Performance in Junior Elite Volleyball Players With Patellar Tendinopathy
Source: Eur J Sport Sci. 2025 Nov 8;25(12):e70080. doi: 10.1002/ejsc.70080 (PMC12595543; doi:10.1002/ejsc.70080)
Supplement: Supplementary file 1 — Supporting Information S1 [file EJSC-25-e70080-s001.docx]

Supplementary material 1 for manuscript “**Patellar tendon properties and unilateral jump performance in junior elite volleyball players with patellar tendinopathy**” (Khair et al.)

**Main effect of sex on tendon properties and jump performance**

Table 1 Main effect of sex on tendon properties and jump performance.

|  | df | F value | P value |
| --- | --- | --- | --- |
| Patellar tendon CSA | 1-22 | 6.18 | p=0.021 |
| SW velocity | 1-22 | 0.38 | p=0.545 |
| Patellar tendon thickness | 1-22 | 3.83 | p=0.063 |
| UIHBP | 1-16 | 1.05 | p=0.320 |
| Countermovement jump | | | |
| Height | 1-17 | 14.05 | p=0.002 |
| Depth | 1-17 | 3.76 | p=0.069 |
| Depth (%height) | 1-17 | 4.91 | p=0.041 |
| Propulsive duration | 1-17 | 0.04 | p=0.970 |
| Unweighting duration | 1-17 | 0.08 | p=0.785 |
| Braking duration | 1-17 | 0.78 | p=0.386 |
| Peak power braking | 1-17 | 52.7 | p=0.044 |
| Peak power propulsive | 1.17 | 10.8 | p=0.004 |
| RSI_mod_ | 1-17 | 9.33 | p=0.007 |
| Drop jump | | | |
| Height | 1-18 | 7.97 | p=0.011 |
| Propulsive duration | 1-18 | 0.13 | p=0.717 |
| Braking duration | 1-18 | 0.13 | p=0.718 |
| Peak power propulsive | 1-18 | 6.85 | p=0.017 |
| Peak power braking | 1-18 | 0.79 | p=0.781 |
| Peak landing force (%body weight) | 1-18 | 0.02 | p=0.891 |
| RSI | 1-18 | 5.14 | p=0.035 |

It should be noted that all durations reported in the table above are relative.
